# Role of Protein Kinase C in Podocytes and Development of Glomerular Damage in Diabetic Nephropathy

**DOI:** 10.3389/fendo.2014.00179

**Published:** 2014-11-05

**Authors:** Beina Teng, Michelle Duong, Irini Tossidou, Xuejiao Yu, Mario Schiffer

**Affiliations:** ^1^Department of Medicine/Nephrology, Hannover Medical School, Hannover, Germany

**Keywords:** proteinuria, diabetic podocytopathy, glomerulosclerosis, nephrin endocytosis, effacement

## Abstract

The early glomerular changes in diabetes include a podocyte phenotype with loss of slit diaphragm proteins, changes in the actin cytoskeleton and foot process architecture. This review focuses on the role of the protein kinase C (PKC) family in podocytes and points out the differential roles of classical, novel, and atypical PKCs in podocytes. Some PKC isoforms are indispensable for proper glomerular development and slit diaphragm maintenance, whereas others might be harmful when activated in the diabetic milieu. Therefore, some might be interesting treatment targets in the early phase of diabetes.

## Introduction

Diabetic nephropathy (DN) is the most common cause of the end-stage renal disease (ESRD) (1). The glomerular changes in DN are characterized by excessive accumulation of extracellular matrix (ECM) with thickening of glomerular and tubular basement membranes and mesangial expansion, which ultimately progress primarily to glomerulosclerosis and secondarily to tubulointerstitial fibrosis. The various cell types involved include glomerular endothelial cells, mesangial cells, podocytes, and tubular epithelia, which are all targets of hyperglycemic injury. However, accumulating evidence suggests that the extent of injury and loss of podocytes is a major prognostic determinant in both, type I and type II DN (2–6). As the terminally differentiated podocytes are believed to play a critical role in maintaining the integrity of the glomerular filtration barrier, effaced podocytes may contribute to the development of albuminuria, a hallmark of DN. Although primarily structure proteins were thought to be the key elements that compose the slit diaphragm initially, it has become clear that the slit diaphragm protein complex is a highly dynamic functional protein complex and is able to initiate cascades of signaling pathways that affect podocyte function (7). More recent data indicate that podocytes express receptors for many circulating hormones and growth factors, which also suggest that a more complex cross-talk between the kidney and other organs affected by diabetes may occur in health and disease (8).

Among various signaling kinases, the protein kinase C (PKC) family seems to play a critical role in the pathogenesis of DN (9). The activation of PKCs in the kidney is a well-known pathway of the diabetic milieu. The PKC family is involved in a variety of signal transduction pathways, cell proliferation, differentiation, cell cycle, and apoptosis. However, the role of PKCs on podocytes in DN has not yet been fully defined. This present review will give an overview of the general role of PKCs and summarize the recent research into the regulatory role of PKCs in podocytes under diabetic conditions.

## PKC Subfamilies and Isoforms

All PKC isoforms contain a highly conserved catalytic domain and a regulatory domain. The catalytic domain consists of several motifs and is essential for the ATP/substrate-binding and catalysis, whereas the N-terminal regulatory domain contains an auto inhibitory pseudo-substrate domain and two discrete membrane targeting modules, C1 and C2. The sequence of pseudo-substrate contains an alanine in place of the serine/threonine phospho-acceptor site, but otherwise resembles a PKC substrate (10).

Protein kinase C isoforms are subdivided into three subfamilies based on differences of structure in their N-terminal regulatory domain. The isoforms α, βI, βII, and γ belong to conventional PKCs (cPKCs). The regulatory domains of cPKCs contain a C1 domain that functions as diacylglycerol (DAG)/phorbol 12-myristat 13-acetat (PMA) binding motif and a C2 domain that binds anionic phospholipids in a calcium-dependent manner (10). The novel PKCs (nPKCsδ, ε, η, and θ) also have two C1 domains and a C2 domain. The nPKC C2 domains lack the critical calcium-coordinating acidic residues, which is the distinct difference between cPKC and nPKC. The nPKCs can be maximally activated by agonists that promote DAG accumulation or by PMA, but they are insensitive to calcium. Atypical PKCs (aPKCs, ζ, and λ/ι) lack a calcium-sensitive C2 domain; however, they contain an atypical C1 domain, which binds PIP3 or ceramide, but not DAG or PMA. The activity of aPKCs is primarily regulated by protein–protein interaction and phosphorylation catalyzed by phosphoinositide-dependent kinase-1 (PDK-1). Although a few PKC isoforms are expressed in a tissue-specific manner, most are ubiquitously expressed.

## Regulation of PKCs

Unless it is post-translationally or co-translationally phosphorylated, PKCs are incapable of being activated by DAG or other cofactors (11, 12). PKC is translated as a catalytically inactive protein, is converted into an active enzyme by an initial phosphate addition and then into a mature form by further phosphorylation (11, 13, 14). In addition to phosphorylation on serine and threonine residues, PKCs also undergo phosphorylation by tyrosine kinases. PKCs are regulated by two sequential and equally critical mechanisms: phosphorylation triggered by PDK-1 and binding to DAG and/or other cofactors such as phosphatidylserine (PS) or phorbol ester (PE). Each mechanism regulates the structure, subcellular localization, and function of PKCs (15).

As a consequence of increased glycolytic flux, chronic hyperglycemia elevates the *de novo* synthesis of DAG and thus leads to an increased activation of DAG dependent classic and novel PKC isoforms in cultured bovine aortic endothelial cells and smooth muscle cells (16). Furthermore, high-glucose induced cellular levels lead to an increased generation of advanced glycation end products (AGEs), which initiate several signaling events by activating PKC, MAP kinase, and transcription factors such as nuclear factor-κB (NF-κB). This would increase the activity of various growth factors, such as TGF-β, and thereby alter expression of ECM proteins (17). In addition, under high-glucose conditions PKC is activated by higher concentrations of reactive oxygen species (ROS) generated following AGE:RAGE (AGE receptor) interactions (15, 18). In turn, the ROS are generated via NADPH-oxidase activated by PKC, ROS/PKC therefore can act in a cyclical manner to activate one another (19).

## Conventional PKCs in Diabetic Nephropathy

Among all the PKC isoforms, the role of PKCα in the pathogenesis of DN has been investigated intensively, and several studies have demonstrated that PKCα-deficient mice show a better outcome after streptozotocin (STZ) induced diabetes with less proteinuria and preserved nephrin expression (20, 21). Studies from our group underline the involvement of PKCα in proteinuria development in DN. The expression of PKCα in podocytes of patients with DN was increased. Mice were treated after STZ-induced diabetes with the synthetic PKCα inhibitor (GÖ6976), which prevented proteinuria development and led to preserved nephrin expression. Furthermore, we could show a central role for PKCα in endocytosis of the slit diaphragm component nephrin (22, 23). Quack et al. further concluded that proteinuria of diabetic mice is as a result of increased endocytosis of nephrin, which is mediated by a complex consisting of PKCα, protein interacting with c kinase-1 (PICK1), and beta-arrestin2. They found rising glucose levels go along with increased binding of beta-arrestin to nephrin *in vitro* as well as in *in vivo*, but only with preceding PKCα phosphorylating activity on nephrin. This fact and the indispensability of PICK1 suggest PKCα and PICK1 as possible drug targets in early stages of DN (23). These studies show that expression of PKCα is regulated by glucose concentration in the external milieu of the podocyte and that PKCα is directly involved in the maintenance of the slit diaphragm. High levels of PKCα in podocytes led to enhanced endocytosis of nephrin and instability of the slit diaphragm.

In addition, Menne and colleagues demonstrated that the high-glucose induced downregulation of nephrin is probably caused by the PKCα mediated reduction of the transcriptional factor Wilms tumor 1 (WT-1), which has previously been described as a directly interacting binding partner of the nephrin promoter (24, 25). These findings are consistent with earlier studies, suggesting one of the PKC isoforms might be pivotal for the regulation of nephrin transcription and expression in podocytes (26), but not for CD2AP and Podocin as they remain at levels similar to those in non-diabetic kidneys (27).

Langham et al. showed that in diabetic patients treated with perindopril, an ACE inhibitor, nephrin levels preserved resembling those of non-diabetic subjects (28) and subsequently could be one factor contributing to the anti-proteinuric effects of ACE inhibitors. Another study suggests that the AGEs, which can be inhibited by aminoguanidine, are also implicated in the downregulation of nephrin in diabetes (29). Interestingly, several groups could demonstrate the reduction of PKC activity under the treatment with ACE inhibitors and aminoguanidine in diabetic subjects that could explain the nephrin protective effects (30, 31).

Most previous studies with specific PKCβ inhibitor ruboxistaurin (LX333531) *in vivo* and *in vitro* indicated that PKCβ isoform is primarily responsible for the high-glucose-induced renal effects in diabetes (32–36). Meier et al. tested this hypothesis by inducing DN in PKCβ deficient mice and did not find a significant preventive effect of PKCβ deficiency on albuminuria. In contrast to non-albuminuric diabetic PKCα^−/−^ mice, the loss of the basement membrane proteoglycan perlecan and the podocyte slit diaphragm protein nephrin were not prevented in the PKCβ^−/−^ mice under diabetic conditions (20, 37). However, the hyperglycemia-induced renal and glomerular hypertrophy as well as increased expression of ECM proteins was reduced in PKCβ deficiency diabetic mice.

In summary, the two important physiological features of DN, renal hypertrophy and albuminuria, are regulated through different PKC isoforms; PKCα is involved in the development of albuminuria and maintenance the glomerular filtration barrier structure, whereas the PKCβ-isoform contributes to hyperglycemia-induced renal fibrosis.

Another study by Menne et al. combined the findings about PKCα and PKCβ and demonstrated that a dual inhibition of both isoforms has a synergistic effect and is capable of preventing the development of experimental DN in streptozocin-induced diabetic mice (38). Blocking both isoforms has a beneficial effect on the development of renal hypertrophy and albuminuria in mice after 8 weeks of diabetes. A pharmacological approach with CGP41252, an inhibitor of PKCα and PKCβ showed that the occurrence of albuminuria could be avoided and preexisting albuminuria could be diminished in both type I and type II diabetic mice (38). But the treatment had little impact on the development of renal hypertrophy. Higher doses treatment also increased mortality.

## Essential Role of Novel and Atypical PKCs

Only little is known about PKCε in renal function, especially about its role in podocytes, although several previous studies showed increased expression and activation of PKCε isoform in experimental DN (39, 40). Meier et al. investigated the functional role of PKCε in renal physiology using PKCε-knockout mice and found a renal phenotype with an elevated occurrence of tubulointerstitial fibrosis and glomerulosclerosis, whereas a systemic profibrotic phenotype was not observed (41). Moreover, they demonstrated an increased level of albuminuria in knockout mice whereas the kidney/body ratio remained normal in comparison to non-diabetic wild type mice, indicating that PKCε is probably less implicated in development of renal hypertrophy. Experiments on diabetic mice further showed that knockout of PKCε can exacerbate the renal phenotype with a significantly increased urinary albumin/creatinine ratio and expression of ECM proteins. These data suggest that a rising level of PKCε has a protective function in kidney injury, rather than inducing profibrotic changes.

The atypical isoforms PKCλ and PKCι are both highly expressed in podocytes. Although it is not clear whether both isotypes perform distinct roles in the cell, aPKCs are well-known to be important in the establishment of cell polarity (42, 43). They form an evolutionarily conserved complex consisting of aPKC and PAR3 and PAR6, two PDZ domain containing scaffold proteins (44). Studies showed that the Par3–Par6–aPKC complex interacts with nephrin–podocin through the direct connection of Par3 to nephrin, an essential structural component for the maintenance of integrity of the glomerular filter as well as for the signal transduction (45). Interestingly, our own studies of the glomerular development in regard to the Par polarity complex and slit diaphragm molecules show that the PAR3–PAR6–aPKCλ complex translocates together with other tight junction proteins like ZO-1 from the apical to the basolateral side of the cell preceding the targeting of slit diaphragm components such as nephrin and podocin to the basal membrane, development of foot processes, and the construction of slit diaphragms (46).

The very recent study of Satoh and colleagues shows the necessity of aPKC in the exocytosis of newly produced nephrin and its localization on the surface of podocytes (47). This finding could also partially explain the diminished nephrin expression in proteinuria patients with diabetes (48). Under high-glucose conditions, activation of aPKC presents a protective effect on the nephrin expression (48).

The activation of aPKC is required for the insulin-induced glucose transport and thus defective aPKC activation in muscle and adipocytes has been shown previously in type II diabetic rats, monkeys, and human beings, which leads to a disturbed glucose uptake into muscle and the whole body glucose transfer (49). The knockout of the other isoform PKCζ has no specific renal phenotype but seems to be able compensate for partial functions of aPKCλ loss as the double-knockout leads to glomerular developmental defects with no secondary foot processes (50). This is most likely because of the regulatory cytoskeletal functions of aPKCs with direct influence on small GTPase activation of aPKCs in podocytes (51). Nevertheless, the role of aPKC in DN still remains unclear.

## PKC Up-Regulated Growth Factor Expression in Diabetic Nephropathy

Podocytes are the major site of vascular endothelial growth factor (VEGF) production in the human kidney (52), and the expression of VEGF is increased in podocytes in diabetic rats and human beings (53, 54). Thus, the up-regulation of VEGF plays a critical role in the progression of DN. Hoshi et al. have shown that under high-glucose conditions, VEGF expression was up-regulated mediated through activation of PKC and extracellular signal-regulated kinase (ERK) in podocytes (55). PKC and ERK are known to regulate activator protein-1 (AP-1) activation (56–58), which promotes the binding of AP-1 to the promoter region of the VEGF gene (59, 60). In addition, AGEs upregulate VEGF mRNA levels through transcription factors such as NF-κB and AP-1. AGEs are also able to activate PKCs, which further increase the expression of VEGF. Although VEGF is required for normal glomerulogenesis and essential for maintenance of glomerular filtration barrier, podocyte-specific overexpression of VEGF_164_ or VEGF_165_ isoform in animals leads to structural and functional renal changes similar to those abnormalities seen in DN, including proteinuria, glomerular hypertrophy, glomerular basement membrane thickening, mesangial expansion, loss of slit diaphragms, and podocyte effacement (61, 62). In DN, the activation of TGF-β1 has been demonstrated to promote podocyte apoptosis and the development of glomerulosclerosis. A reduced expression level of the profibrotic cytokine TGF-β1 was detected in diabetic PKCβ^−/−^ mice, while the alteration was not observed in diabetic PKCα^−/−^ mice (20). This observation suggested that PKCβ isoform is more important in the up-regulation of TGF-β and for the development of glomerular fibrosis under diabetic conditions, whereas PKCα shows its critical role in the integrity of glomerular filtration barrier (37). However, PKCα plays a key role in the signaling response after stimulation with TGF-β1. An enhanced and prolonged activation of PI3K/AKT and ERK1/2 as well as a reduced proapoptotic signaling via p38MAPK in PKCα-knockout podocytes compared to wild type podocytes was detected after TGF-β1 treatment, which indicated the involvement of PKCα in the TGF-β mediated apoptosis (63).

Of note, deletion of PKCε signaling not only leads to increased expression of TGF-β1 but also induces activation of the TGF-β1 signaling pathway in glomeruli (41). PKCι might also mediate a high-glucose-induced increase in TGF-β receptor II (TGF-βRII) promoter activity, which leads to the up-regulation of TGF-βRII and fibronectin (64).

## PKC Regulated Structure Proteins in Podocytes

P-cadherin as member of the classical cadherins, a superfamily of glycoproteins, is known to be a basic scaffold for the glomerular slit diaphragm (65). Xu et al. have first demonstrated decreased expression of P-cadherin mRNA and protein in experimental diabetic glomeruli and in high-glucose stimulated podocytes, which suggests that a potential role for P-cadherin loss in the development of proteinuria in early DN (66). They also found that PKC inhibitors could ameliorate the decrement of P-cadherin in podocytes under high-glucose conditions. Thus, activation of PKC regulated pathways seems to be involved in the regulation of P-cadherin expression and contributes to the disruption of podocyte integrity (66).

Nevertheless, it seems likely that the molecular changes of the slit diaphragm complex, but not one single slit diaphragm-associated molecule contribute to the pathogenesis of glomerular filtration barrier in DN. P-cadherin, α-, β-, and γ-catenin, and ZO-1 are described to compose adherens junctions at the slit diaphragm and establish the link with the actin cytoskeleton (65, 67). However, the role and regulation of β-catenin with P-cadherin and actin cytoskeletal proteins have not been thoroughly explored. In the situation of the podocyte injury induced by high glucose, β-catenin will be released from the destruction complex because of the activation of the Wnt-pathway. Afterwards β-catenin translocates into the nucleus to activate the downstream genes via the aggregation of a transcriptional complex with TCF/LEF, which leads to proteinuria and glomerulosclerosis (68, 69). Several groups demonstrated that PKC activation phosphorylated N-terminal serine residues of β-catenin, which promoted β-catenin degradation (70, 71). In contrast, our ongoing work was focused on several PKC isoforms, whose activation is able to dephosphorylate β-catenin to prevent β-catenin from degradation. It is still unknown, whether this process is protective for the high-glucose-induced cell adherens junction reduction. Nevertheless, PKC activation may be a novel mechanism in regulating β-catenin in glomerular injury, which will be further investigated by our laboratory.

## Conclusion

**Figure 1:**
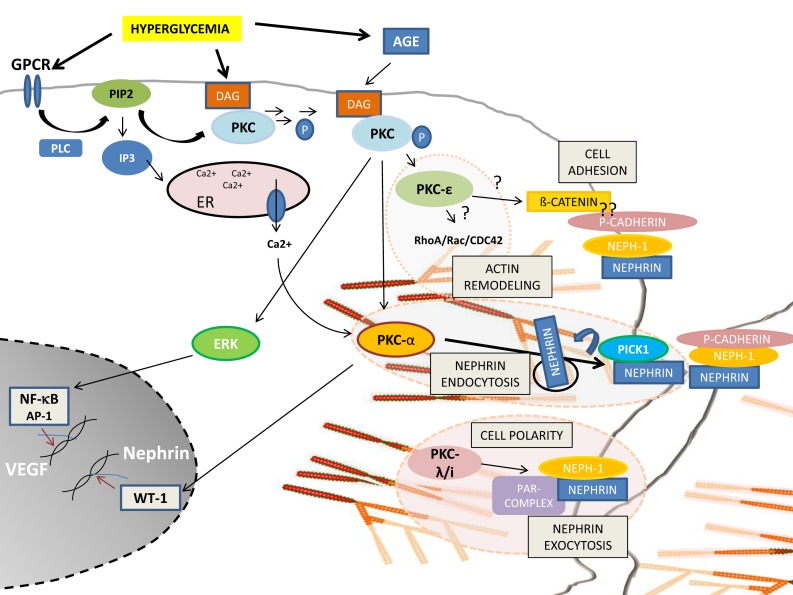
**Schematic overview of PKC-isoform functions in podocytes**. DAG or calcium activated PKCα induces nephrin endocytosis via the adaptor molecule Pick1. PKCλ/ι is required for foot process formation, cell polarity, and nephrin exocytosis. PKCε might be involved in actin remodeling via small GTPases and might orchestrate cell adhesion and cell–cell contact formation.

Podocyte injury or podocyte loss is a hallmark for the pathogenesis of DN. Based on the above described findings, PKC activation seems to be the most critical pathway involved in the progression of glomerular injury. Obviously, there is a fine balance between activation and inactivation of the different PKC isoforms and their cross-talk involving foot process cytoskeletal architecture, cellular junction formation, and orchestration of turnover and surface expression of slit diaphragm components (Figure 1). Therefore, PKCs and their involved pathways are potential therapeutic targets in podocytes to prevent the progression of diabetic glomerulopathy.

## Conflict of Interest Statement

The authors declare that the research was conducted in the absence of any commercial or financial relationships that could be construed as a potential conflict of interest.
